# Improved Glycaemic Control and Nephroprotective Effects of Empagliflozin and Paricalcitol Co-Therapy in Mice with Type 2 Diabetes Mellitus

**DOI:** 10.3390/ijms242417380

**Published:** 2023-12-12

**Authors:** Abdulrahman Mujalli, Wesam F. Farrash, Ahmad A. Obaid, Anmar A. Khan, Riyad A. Almaimani, Shakir Idris, Mohamed E. Elzubier, Elshiekh Babiker A. Khidir, Akhmed Aslam, Faisal Minshawi, Mohammad A. Alobaidy, Adel B. Alharbi, Hussain A. Almasmoum, Mazen Ghaith, Khalid Alqethami, Bassem Refaat

**Affiliations:** 1 Department of Clinical Laboratory Sciences, Faculty of Applied Medical Sciences, Umm Al-Qura University, Al Abdeyah, Makkah P.O. Box 7607, Saudi Arabia; 2 Biochemistry Department, Faculty of Medicine, Umm Al-Qura University, Al Abdeyah, Makkah P.O. Box 7607, Saudi Arabia; 3 Department of Anatomy, Faculty of Medicine, Umm AlQura University, Makkah P.O. Box 7607, Saudi Arabia; 4 Department of Laboratory, Al-Noor Specialist Hospital, Makkah P.O. Box 7607, Saudi Arabia

**Keywords:** SGLT2 inhibitor, adipokines, INOS, KIM-1, NGAL, TGF-β, vitamin D

## Abstract

Herein, we measured the antidiabetic and nephroprotective effects of the sodium–glucose cotransporter-2 inhibitor (empagliflozin; SGLT2i) and synthetic active vitamin D (paricalcitol; Pcal) mono- and co-therapy against diabetic nephropathy (DN). Fifty mice were assigned into negative (NC) and positive (PC) control, SGLT2i, Pcal, and SGLT2i+Pcal groups. Following establishment of DN, SGLT2i (5.1 mg/kg/day) and/or Pcal (0.5 µg/kg/day) were used in the designated groups (5 times/week/day). DN was affirmed in the PC group by hyperglycaemia, dyslipidaemia, polyuria, proteinuria, elevated urine protein/creatinine ratio, and abnormal renal biochemical parameters. Renal SREBP-1 lipogenic molecule, adipokines (leptin/resistin), pro-oxidant (MDA/H_2_O_2_), pro-inflammatory (IL1β/IL6/TNF-α), tissue damage (iNOS/TGF-β1/NGAL/KIM-1), and apoptosis (TUNEL/Caspase-3) markers also increased in the PC group. In contrast, renal lipolytic (PPARα/PPARγ), adiponectin, antioxidant (GSH/GPx1/SOD1/CAT), and anti-inflammatory (IL10) molecules decreased in the PC group. Both monotherapies increased insulin levels and mitigated hyperglycaemia, dyslipidaemia, renal and urine biochemical profiles alongside renal lipid regulatory molecules, inflammation, and oxidative stress. While SGLT2i monotherapy showed superior effects to Pcal, their combination demonstrated enhanced remedial actions related to metabolic control alongside renal oxidative stress, inflammation, and apoptosis. In conclusion, SGLT2i was better than Pcal monotherapy against DN, and their combination revealed better nephroprotection, plausibly by enhanced glycaemic control with boosted renal antioxidative and anti-inflammatory mechanisms.

## 1. Introduction

Diabetic nephropathy (DN) is a major cause of end-stage kidney disease and chronic renal failure, which is clinically characterised by albuminuria and reduced glomerular filtration rate (GFR) [1]. During high-glucose condition, renal glucose reabsorption also increases due to upregulations in tubular sodium–glucose cotransporter-2 (SGLT2) and glucose transporter-2 (GLUT2) proteins [2,3]. Moreover, glomerular and tubular damage are linked with higher expression of neutrophil gelatinase-associated lipocalin (NGAL) and kidney injury molecule-1, respectively [4].

The pathogenesis of DN is predominantly driven by augmented production of reactive oxygen (ROS) and nitrogen (RNS) species due to upregulated inducible nitric oxide synthase (iNOS) enzyme and induction of mitochondrial damage, thus causing renal injury [5,6]. In addition, hyperglycaemia and dyslipidaemia perturbs renal antioxidant capacity by lowering glutathione (GSH), glutathione peroxidase-1 (GPx1), superoxide dismutase-1 (SOD1), and catalase (CAT) that subsequently induce lipid peroxidation, protein damage, and chronic inflammation [5,6,7]. Diabetes also triggers renal steatosis and lipotoxicity via inhibiting the peroxisome proliferator-activated receptor (PPAR)-α and -γ lipolytic [8,9], whilst enhancing the sterol regulatory element-binding protein-1 (SREBP-1) lipogenic molecules [10,11]. Furthermore, hyperglycaemia and dyslipidaemia exacerbate renal inflammation by increasing the production of pro-inflammatory cytokines, such as interleukin (IL)-1β, IL-6, and tumour necrosis factor (TNF)-α, while reducing the anti-inflammatory cytokine, IL-10 [12,13]. Renal steatosis also reduces adiponectin [14], and elevates leptin [15] and resistin production in renal tissues [16]. Persistent oxidative stress and inflammation then promote cell apoptosis by upregulating transforming growth factor-β (TGF-β) with caspase-3 (Casp-3), thus aggravating glomerular and tubular damage [17,18].

The inhibitors of SGLT2 (SGLT2i) are novel antidiabetic agents that reduce blood glucose levels by simultaneously promoting glucosuria and natriuresis [19]. More recently, numerous experimental and clinical studies have reported that different analogues of SGLT2i delayed the onset and progression of DN, mainly by reducing oxidative stress and inflammation, whilst improving glomerular filtration [20,21,22,23]. Similarly, the synthetic analogue of active vitamin D (VD3), paricalcitol (Pcal; 19-nor-1α-25-2(OH) D2), is mainly used to treat hyperparathyroidism associated with chronic kidney disease. Others, however, disclosed antidiabetic and nephroprotective actions for Pcal, both in animals [24,25,26] and humans [24,27,28] by reducing inflammation and boosting antioxidants in renal tissues [26,29].

Despite the previous reports, the potential for synergy between SGLT2i and Pcal in the treatment of DN remains elusive. Hence, this study aimed to validate our hypothesis that SGLT2i and Pcal co-therapy could provide a more effective nephroprotective approach against DN, by achieving enhanced glycaemic control, and potentiated antioxidant and anti-inflammatory effects.

## 2. Results

### 2.1. Metabolic and Renal Biochemicals Profiles

The PC group showed significantly lower body weight with drastic elevations in serum concentrations of FBG, total cholesterol, LDL, and TG that coincided with marked declines in serum insulin, total protein, albumin, and HDL together with urine Cr levels, compared with the NC mice (Table 1; *p* < 0.001 for all markers). Moreover, serum urea and Cr levels alongside spot urine total protein concentrations and protein/Cr ratio were markedly higher in the PC than the NC group (Table 1; *p* < 0.001 for all markers). While both monotherapies increased the body weight and ameliorated the metabolic and renal biochemical markers relative to PC animals, the effects of the SGLT2i single therapy were significantly more pronounced than the Pcal group. However, all markers remained abnormal in both monotherapies in comparison to the NC group. On the other hand, the best ameliorative actions were detected with the dual therapy protocol relative to the PC and both monotherapy groups (Table 1; *p* < 0.01).

### 2.2. Markers of Renal Tissue Damage

**Figure 1:**
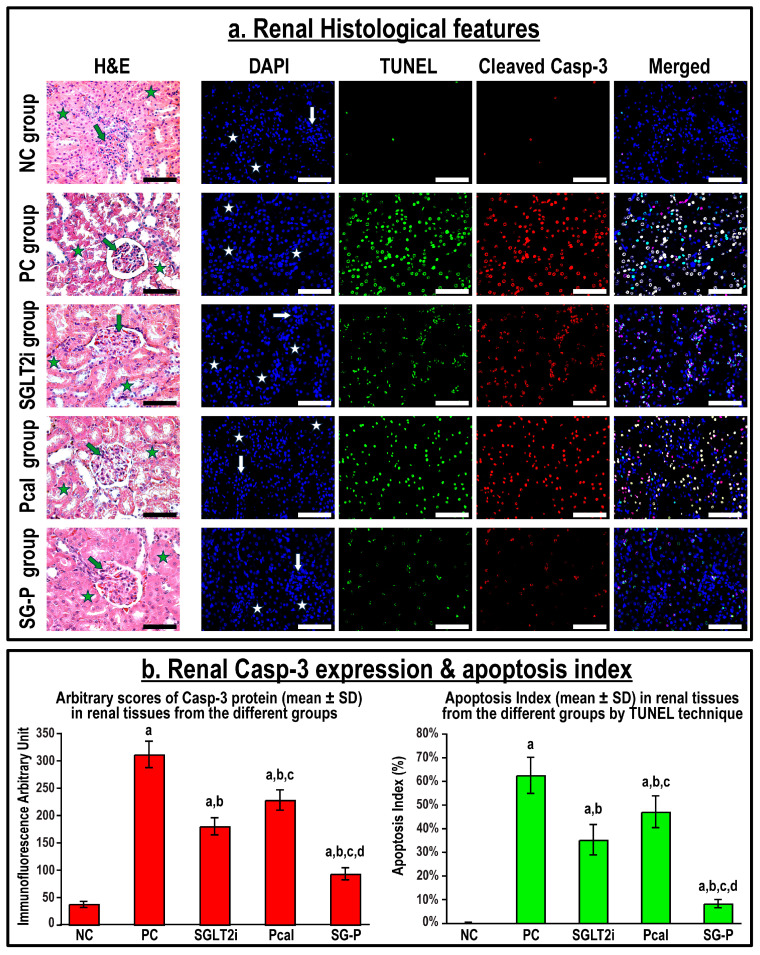
(**a**) Histology of renal tissue by H&E (green arrow = glomerulus; green star = renal tubule) alongside immunofluorescence co-localisation of apoptotic bodies by the TUNEL technique (green) and cleaved Casp-3 protein (red), and counterstaining with DAPI in renal tissues from all groups (40× objective; scale bar = 10 µm; white arrow = glomerulus; white star = renal tubule). (**b**) Relative protein expression of Casp-3 and percentage of apoptosis in renal tissues, from all groups, are shown as graph bars (data represent mean ± SD; a = *p* < 0.05 compared with the NC group; b = *p* < 0.05 compared with the PC group, c = *p* < 0.05 compared with the SGLT2i, and d = *p* < 0.05 compared with the Pcal group).

**Figure 2:**
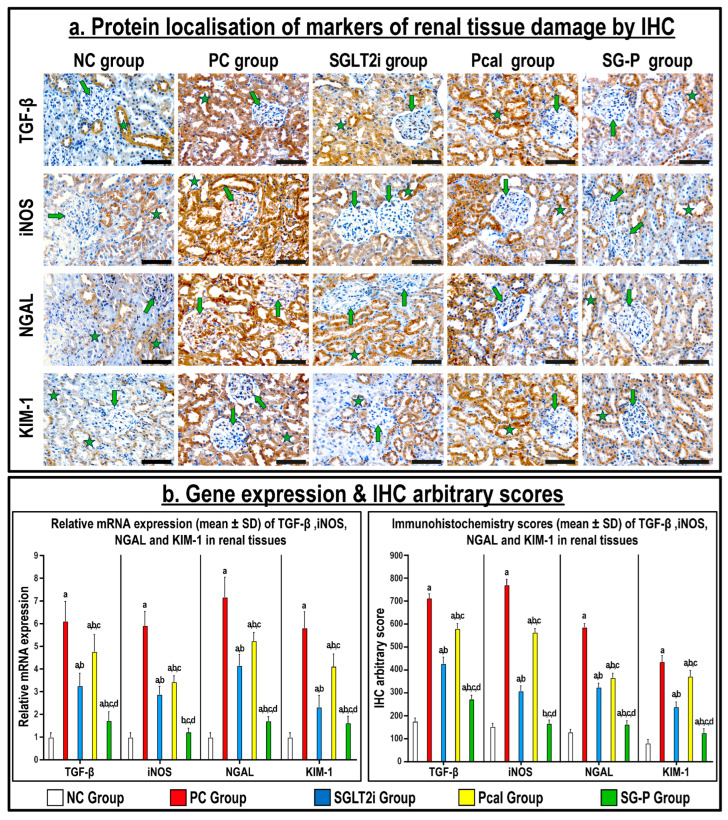
(**a**) Immunohistochemistry (IHC) localisation of TGF-β, iNOS, NGAL, and KIM-1 in renal tissues (40× objective; scale bar = 10 µm; green arrow = glomerulus; green star = renal tubule) together with (**b**) their relative mRNA expression and IHC arbitrary scores, in all groups, are shown as graph bars (data represent mean ± SD; a = *p* < 0.05 compared with the NC group; b = *p* < 0.05 compared with the PC group, c = *p* < 0.05 compared with the SGLT2i, and d = *p* < 0.05 compared with the Pcal group).

The renal tissue from the NC group displayed normal histology by H&E with scarce numbers of apoptotic bodies by the TUNEL technique, and low protein expression of cleaved Casp-3 by immunofluorescence (Figure 1). In contrast, significant glomerular and tubular damages were observed in the PC renal specimens that were portrayed by cupping and widening of glomerular capsules, fragmentation of tubules with protrusion of nuclei, and with significant increase in the number of apoptotic cells with increased Casp-3 protein expression than the NC group (*p* < 0.001 for both markers). Additionally, the gene and protein expression of TGF-β, iNOS, NGAL, and KIM-1 increased substantially in the PC renal tissues compared to the NC specimens (Figure 2; *p* < 0.001 for all markers). Single treatment with SGLT2i and Pcal improved renal histopathological features, reduced the percentage of apoptotic cells, and the expression of Casp-3 (Figure 1), as well as the gene and protein expression of TGF-β, iNOS, NGAL, and KIM-1 (Figure 2) relative to the PC group, with better impact of the SGLT2i than the Pcal monotherapy. However, co-treatment with SGLT2i and Pcal showed the best improvements related to cell survival together with the lowest expression of all tested markers of renal tissue damage (Figure 1 and Figure 2).

### 2.3. Renal Metabolic Regulatory Molecules

#### 2.3.1. Renal Glucose Transporting Proteins

**Figure 3:**
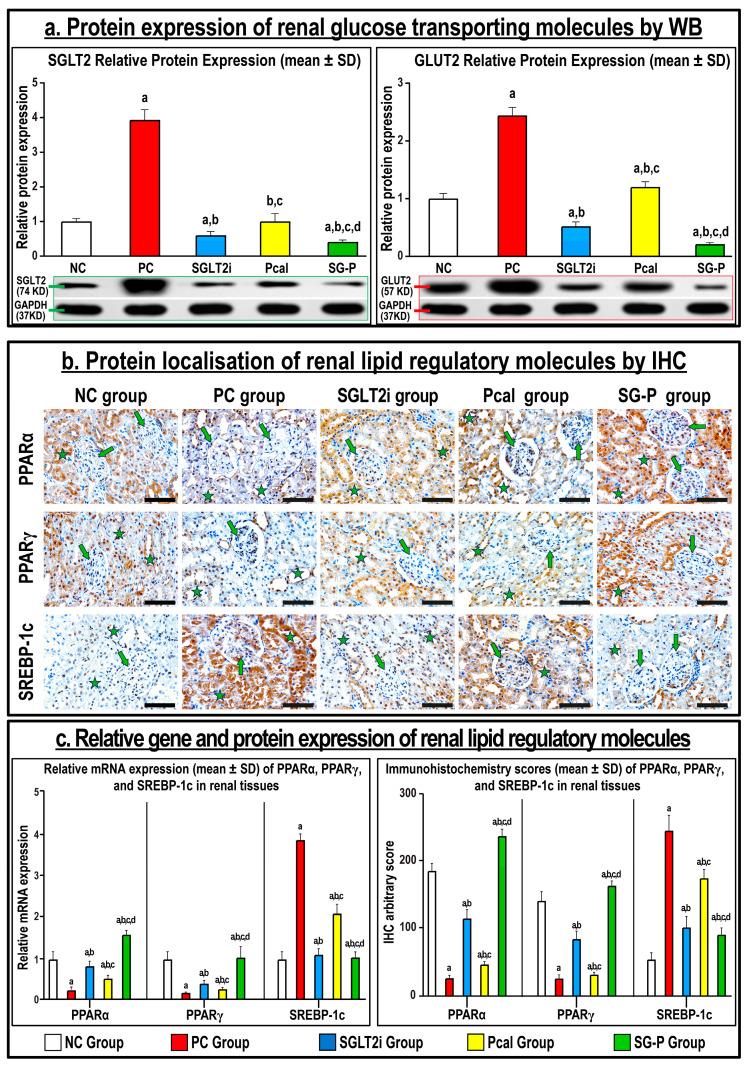
(**a**) Expression of SGLT2 and GLUT2 proteins in renal tissue homogenates from all groups by Western blotting and their relative expression were normalised with GAPDH protein and shown as graph bars (mean ± SD; a = *p* < 0.05 compared with the NC group; b = *p* < 0.05 compared with the PC group, c = *p* < 0.05 compared with the SGLT2i, and d = *p* < 0.05 compared with the Pcal group). (**b**) Immunohistochemistry (IHC) localisation of PPARα, PPARγ, and SREBP-1c proteins in renal tissues (40× objective; scale bar = 10 µm; green arrow = glomerulus; green star = renal tubule) alongside (**c**) their relative mRNA expression and IHC scores in the different groups are shown as graph bars (data is shown as mean ± SD; a = *p* < 0.05 compared with the NC group; b = *p* < 0.05 compared with the PC group, c = *p* < 0.05 compared with the SGLT2i, and d = *p* < 0.05 compared with the Pcal group).

SGLT2 and GLUT2 protein expression by Western blotting was substantially higher in the PC relative to the NC renal specimens (Figure 3a; *p* < 0.001 for both proteins). While both monotherapies significantly lowered the expression of both proteins compared with the PC group, the levels were markedly lower in the SGLT2i treatment, whereas the Pcal group showed equal expression, relative to the NC renal tissue. Nonetheless, the minimal expression of SGLT2 and GLUT2 proteins were seen in the dual therapy protocol in comparison with all groups (Figure 3a).

#### 2.3.2. Renal Lipid Regulatory Molecules

The gene and protein expression of PPARα and PPARγ decreased, whilst SREBP-1c levels increased, in the PC compared with the normal group (Figure 3b,c; *p* < 0.001 for all markers). Treatment with SGLT2i or Pcal augmented the mRNAs and proteins of PPARα and PPARγ, whereas it lowered those of SREBP-1c, relative to the PC group. Although the co-treatment approach further decreased the gene and protein expression of PPARα and PPARγ alongside increased SREBP-1c relative to both monotherapies, the levels of all molecules remained significantly abnormal compared with the NC group (Figure 3a,b).

#### 2.3.3. Renal Tissue Concentrations of Adipokines

**Figure 4:**
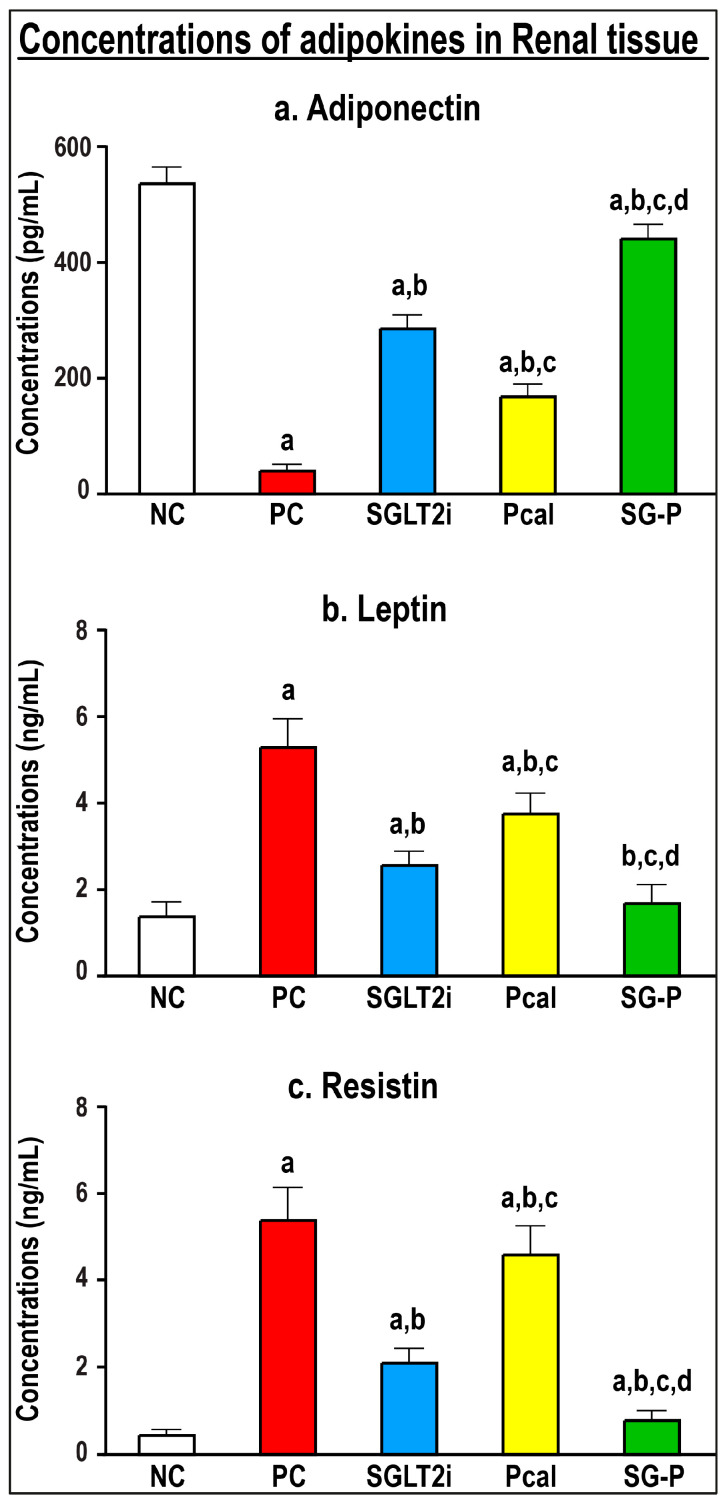
Renal tissue concentrations (mean ± SD) of (**a**) adiponectin, (**b**) leptin, and (**c**) resistin in the different study groups are displayed as graph bars (a = *p* < 0.05 compared with the NC group; b = *p* < 0.05 compared with the PC group, c = *p* < 0.05 compared with the Pcal group, and d = *p* < 0.05 compared with the OM group).

Renal tissue concentrations of adiponectin (Figure 4a) diminished, whilst leptin (Figure 4b) and resistin (Figure 4c) increased, drastically relative to the NC renal specimens. Both monotherapy protocols reduced leptin and resistin alongside elevated adiponectin concentrations in renal tissues compared with the PC mice, and SGLT2i revealed better actions than Pcal monotherapy. On the other hand, the combined treatment regimen exhibited the best alleviatory effects on all tested adipokines relative to the PC and monotherapies (Figure 4).

### 2.4. Renal Tissue Concentrations of Inflammatory and Oxidative Stress Markers

Concentrations of TNF-α, IL-1β, IL6, MDA, and H_2_O_2_ augmented, whilst IL10, GSH, GPx1, SOD1, and CAT declined significantly in the PC renal tissue lysates relative to the NC group (Table 2). The amounts of pro-inflammatory and pro-oxidative stress markers decreased, whilst those of anti-inflammatory cytokines and antioxidative stress molecules increased substantially in both monotherapy groups compared with the PC renal tissues. Nonetheless, all molecules were markedly different in both monotherapy groups compared with the NC mice (Table 2). Additionally, the concentrations of the tested inflammatory and oxidative stress molecules were equal between the SGLT2i and Pcal groups, except for IL-1β and IL6, which were significantly lower in the latter group. On the other hand, the co-treatment protocol showed the lowest levels of pro-inflammatory and oxidative stress molecules together with the highest amounts of anti-inflammatory and antioxidant markers, in comparison with the PC, and both monotherapy groups. Nevertheless, the concentrations of all cytokines and oxidative stress markers were markedly abnormal in the SG-P group compared with the NC group (Table 2).

## 3. Discussion

The present study investigated the potential mitigating actions of SGLT2i and/or Pcal single and dual therapies against diabetic nephropathy. In the PC group, DN was confirmed by high FBG, decreased serum insulin, abnormal lipid profile, hypoproteinaemia, increased serum creatinine and urea levels alongside proteinuria, low urine creatinine levels, and elevated urine protein/Cr ratio. The PC renal tissues also showed marked increases in SGLT2, GLUT2, iNOS, SREBP-1, TGF-β, NGAL, KIM-1, and Casp-3 expression alongside a substantial increase in the numbers of apoptotic cells compared to the NC group. Levels of TNF-α, IL-1β, IL-6, leptin, resistin, MDA, and H_2_O_2_ also increased markedly in the PC renal tissues, whereas PPARα, PPARγ, IL-10, adiponectin, GSH, SOD1, CAT, and GPx1 declined relative to the NC group.

In agreement with our data, renal tubular cells increase their glucose reabsorption capacity by upregulating SGLT2 and GLUT2 transporting proteins during hyperglycaemic states [2,3]. Renal iNOS expression also increases with chronic hyperglycaemia and incites mitochondrial dysfunction, which then promotes renal oxidative stress due to ROS and RNS overproduction alongside declines in many antioxidants, including GPx1, SOD1, CAT, and GSH [5,6,7,30,31,32]. Moreover, DM triggers renal steatosis and lipotoxicity by inhibiting PPARα and PPARγ lipolytic [8,9,33,34], whilst increasing SREBP-1 lipogenic molecules [10,11], which then provoke chronic renal inflammation by augmenting renal TNF-α, IL-1β, and IL-6 levels, and reducing the potent anti-inflammatory cytokine, IL-10 [12,13]. The levels of several renal adipokines are likewise altered in DN, and renal inflammation is aggravated by a substantial decrease in adiponectin [14,35] that coincides with increases in leptin [15,36] and resistin [16,37] levels. Chronic renal oxidative stress and inflammation subsequently trigger apoptosis through TGF-β-mediated activation of Casp-3 [17,18], with glomerulopathy and tubular damage that are manifested by albuminuria and decreased GFR [38,39], and by increasing renal expression of NGAL and KIM-1 [4,40]. Together, our results and many earlier reports, advocate that the pathomechanisms underlying DN are intricate and involve a compensatory increase in glucose reabsorption that induces renal metabolic reprograming followed by renal steatosis, mitochondrial damage, oxidative stress, inflammation, and cell death [4,8,9,10,11,17,18,33,34,38,39,40].

Many studies have reported renoprotective effects for the new anti-diabetic drugs, SGLT2i, that counteract hyperglycaemia by simultaneously promoting glucosuria and natriuresis [20,21,22]. In detail, treating diabetic mice with SGLT2i attenuated hyperglycaemia, dyslipidaemia, renal lipotoxicity, glomerular damage, and albuminuria, as well as increased renal PPARα and PPARγ with concurrent decreases in several inflammatory and oxidative stress markers [41,42,43,44]. SGLT2i also alleviated proteinuria and inflammation alongside lowered renal NGAL and KIM-1 expression, whilst promoting SOD1 and GSH levels in diabetic rats [45,46]. Others have also shown that SGLT2i increased β-cell mass and insulin production by simultaneously inducing proliferation and inhibiting apoptosis in the pancreas of diabetic animals [47,48]. In clinical settings, SGLT2i also reduced albuminuria, modulated the levels of adipokines, and slowed the progression of chronic kidney disease [15,20,21,22]. On the other hand, the synthetic analogue of active VD_3_, Pcal, also exerted antidiabetic actions by increasing insulin production following impedance of oxidative stress and inflammation of pancreatic β-cells in rats injected with STZ [49]. Additionally, Pcal decreased TNF-α, IL-1β, IL-6, TGF-β, NGAL, KIM-1, and Casp-3, whilst enhancing the expression of antioxidants and IL-10 in renal tissues, thus ameliorating DN in murine rodents [24,25,26]. Several clinical studies have likewise reported that Pcal treatment lowered NGAL and several pro-inflammatory cytokines in chronic kidney disease patients [29], and the drug also reduced albuminuria and halted the onset and progression of nephropathy in diabetic patients [24,27,28].

In this study, both SGLT2i and Pcal single therapies markedly reduced FBG, increased serum insulin, and ameliorated proteinuria with serum lipid and renal biochemical profiles relative to the PC group. SGLT2i and Pcal monotherapy groups also revealed better renal histology with significantly lower apoptosis index and decreased Casp-3 gene and protein expression compared to the PC renal specimens. Renal concentrations of antioxidants, IL-10, and adiponectin increased, whilst the oxidative stress markers, proinflammatory cytokines, leptin, resistin, TGF-β, iNOS, NGAL, and KIM-1 declined with both monotherapies compared to the PC animals. Our findings are aligned with many reports that have underscored the beneficial nephroprotective effects of SGLT2i [15,20,21,22] and Pcal [24,27,28,29] that could include glycaemic control with inhibition of renal lipotoxicity, oxidative stress, and inflammation.

SGLT2i monotherapy, however, demonstrated significantly fewer numbers of dead cells and lower expression of iNOS, TGF-β, Casp-3, NGAL, and KIM-1 relative to the Pcal group. Renal SGLT2 and GLUT2 protein expression was also markedly lower in the SGLT2i than the Pcal group. Our results denote more efficient renoprotective actions for SGLT2i against DN, which could be related to its dual glycosuric and natriuretic actions that may provide better controls of blood glucose levels and renal haemodynamics [20,21,22]. Another explanation could be that SGLT2i has stronger modulatory effects than Pcal on renal adipokines and lipogenic pathways. In this context, SGLT2i alleviated DN in experimental and clinical studies by diminishing the expression of SREBP-1 lipogenic molecule with the levels of pathogenic adipokines, leptin and resistin, whilst increasing adiponectin levels and promoting the expression of PPARα and PPARγ lipolytic molecules, thus inhibiting renal lipotoxicity, oxidative stress, and inflammation [15,20,43,44]. Although Pcal therapy elevated cardiac adiponectin levels in apolipoprotein-E deficient mice [50], it showed limited effects on hepatic adiponectin and leptin levels, as well as the expression of their receptors, PPARα, PPARγ, and SREBP-1 in rats with non-alcoholic fatty liver disease [51]. Accordingly, we hypothesise that SGLT2i could be a more efficacious treatment for DN than Pcal by exerting superior glycaemic control with modulation of renal adipokines and lipogenic pathways, thereby minimising renal steatosis, oxidative stress, and inflammation [15,20,21,22,43,44]. Nonetheless, further studies are warranted to compare the effects of SGLT2i and Pcal on urine glucose concentrations, renal lipid metabolism, and renal haemodynamics to support our hypothesis.

To the best of our knowledge, this study is the first to evaluate the antidiabetic and renoprotective effects of SGLT2i and Pcal dual therapy. Our results showed boosted glycaemic and lipidemic control and marked reductions in proteinuria with renal oxidative stress, inflammation, and apoptosis relative to the PC and both monotherapy groups. The co-therapy regimen was also associated with a substantial decrease in SGLT2 and GLUT2 protein expression compared with the NC, PC, and both monotherapy groups. Hence, we postulate that the superior glycaemic control and nephroprotective outcomes achieved with SGLT2i and Pcal dual therapy could be attributed to enhanced glucosuria mediated by more potent inhibition of renal glucose reabsorption, thus alleviating renal lipotoxicity, oxidative stress, inflammation, and cellular apoptosis [20,21,22,24,27,28]. However, future studies should measure the effects of SGLT2i and/or Pcal single and dual therapies on pancreatic β-cell function alongside renal glucose regulatory molecules to corroborate our proposal.

There are several drawbacks to the current study. First, we did not measure urine concentrations of ketone bodies, as well as the effects of adding Pcal with SGLT2i on ketoacidosis, which is a potential serious complication of SGLT2i therapy [52,53]. Moreover, SGLT2i treatment alters renal handling of mineral homeostasis, thus increasing parathyroid hormone with hyperphosphatemia alongside promoting calcinuria [54,55]. Hence, future studies should measure urine concentrations of ketone bodies and calcium together with serum levels of calciotropic hormones with SGLT2i and/or Pcal treatments to precisely determine their effects on diabetic ketoacidosis [52,53] and bone health [54,55]. Although both SGLT2i [47,48] and Pcal [49] improved insulin production and secretion in the pancreas of diabetic murine by attenuating inflammation and oxidative stress, the present study did not include pancreatic specimens. Therefore, future studies should concurrently measure the protective effects of single and dual therapies in pancreatic and renal tissues.

## 4. Materials and Methods

### 4.1. Ethical Statement

Ethical approval (#HAPO-02-K-012-2022-11-1340) for all animal experiments was obtained from The Biomedical Research Ethics Committee at Umm Al-Qura University, Makkah, Saudi Arabia.

### 4.2. Drugs

Streptozotocin (STZ) ≥ 98% purity (Sigma-Aldrich Co., St. Louis, MO, USA), the inhibitor of sodium–glucose cotransporter-2 (SGLT2i; empagliflozin (Jardiance^®^; Boehringer Ingelheim Limited, Auckland, New Zealand), and paricalcitol (Pcal; Zemplar™; AbbVie Inc., North Chicago, IL, USA) were used.

### 4.3. Induction of Diabetic Nephropathy and Treatment Protocols

**Figure 5:**
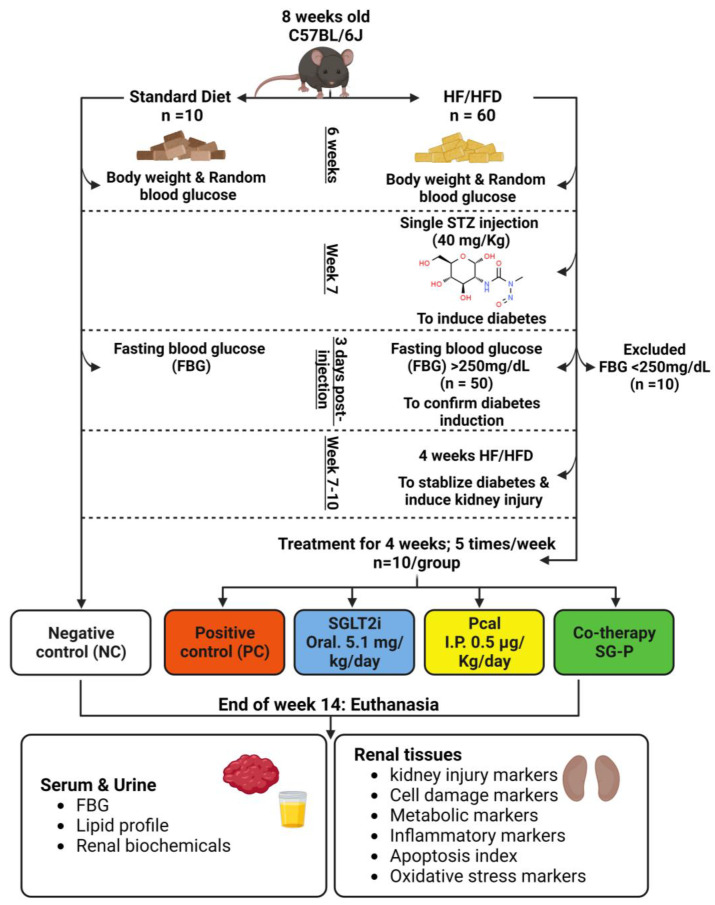
Summary of study design and workflow. Adult male mice (*n* = 60) were used, and while the negative control group mice (*n* = 10) were fed with normal laboratory diet throughout the study, the remaining mice received high-fructose/high-fat diet (HF/HFD) for six weeks. At week 7, a single dose of streptozotocin (STZ; 40 mg/kg) was injected to all animals, except for the NC group. Development of diabetes mellitus was confirmed in 50 mice by elevated fasting blood glucose (FBG; >250 mg/dL). The diabetic mice then continued HF/HFD for another four weeks. Treatments with sodium–glucose cotransporter-2 inhibitor (SGLT2i; 5.1 mg/kg/day; 5 times/week), paricalcitol (Pcal; 0.5 µg/kg/day; 5 times/week) single and dual therapies were initiated at week-11 and lasted for four weeks. Serum metabolic and renal biochemical profiles alongside the expression of several renal markers of tissue damage and metabolic pathways were investigated at the end of the study to measure and compare the remedial effects of single and dual therapies against diabetic nephropathy.

Sixty male wild-type C57BL/6J mice of 8 weeks of age and weighing between 20–25 g body weight were used following one week of acclimatisation. All animals were housed in a temperature-controlled room with a 12 h light/dark cycle (*n* = 5 mice/cage). The total study duration was 14 weeks and included 10 weeks for establishing diabetic nephropathy (DN) followed by four weeks of treatment (Figure 5). The negative control (NC) group (*n* = 10) received standard laboratory chow (5% fat, 45% carbohydrate, and 21% protein) throughout the study, whilst the remaining 50 animals were fed for six weeks with freshly prepared high-fructose/high-fat diet (HF/HFD) by adding 10% fat to the standard diet alongside drinking water containing 20% fructose to induce obesity and insulin resistance, as previously described [1]. Physical appearance, body weight, and random blood glucose levels were recoded weekly to confirm development of obesity and insulin resistance. A single dose of STZ (40 mg/kg) was dissolved in 0.1 M citrate buffer (pH 4.5) and injected intraperitoneally after overnight fasting in the animals that received HF/HFD (*n* = 50) to mimic type 2 diabetes mellitus (T2DM), as reported earlier [45,56]. The mice then received oral glucose solution (10% *w*/*v*) during the first 24 h post-injection to prevent STZ-induced hypoglycaemia. Fasting blood glucose (FBG) levels were measured three days post-STZ injection by an Accu-Chek glucometer (Roche Diabetes Care, Inc., Indianapolis, IN, USA), and T2DM was assured when the levels exceeded 250 mg/dL. While mice with FBG < 250 mg/dL were excluded (*n* = 10) to avoid spontaneous recovery to normoglycaemia [57], the remaining 40 mice with confirmed hyperglycaemia (>250 mg/dL) received another cycle of HF/HFD for four weeks to ensure T2DM-induced kidney injury.

The diabetic mice were then distributed equally (10 mice/group) into the following: the positive (PC) control, SGLT2i and paricalcitol (Pcal) single therapies, and the co-treatment group (SG-P) that received SGLT2i with Pcal simultaneously. Freshly prepared oral SGLT2i (5.1 mg/kg/day; 5 times/week) and/or Pcal intraperitoneal injections (0.5 µg/kg/day; 5 times/week) were given to the assigned groups for four weeks. According to the dose conversion equation between human and mouse [58], the applied doses of SGLT2i and Pcal were equivalent to the recommended maximal daily doses for and adult human of 60 kg body weight (SGLT2i: 25 mg/day; 0.42 mg/kg/day and Pcal: 2.4 µg/day; 0.04 µg/kg/day) [59,60]. Moreover, the therapeutic doses used, and duration were constant with many earlier reports having demonstrated no toxicological side effects in their animal studies [24,61].

### 4.4. Samples Collection and Processing

After fasting for 12 h at the end of the study, a spot urine sample was collected by an insulin syringe from the urinary bladder, whilst a blood sample was drawn from the retro-orbital plexus of anaesthetized mice. The urine samples collected were then centrifuged (6000× *g*) at 4 °C for 20 min and the supernatants were stored at −80 °C, whereas serum samples were stored at −20 °C, until used. Both kidneys were dissected from each mouse with one part being processed by traditional histopathology methods prior to embedding intro paraffin blocks. Another renal specimen (50 mg) from each mouse was used for total RNA extraction by a PureLink™ RNA Mini Kit (Thermo Fisher Scientific, Emeryville, CA, USA) followed by cDNA synthesis using a high-capacity Reverse Transcription Kit (Thermo Fisher Scientific). Total protein was also extracted from a third renal sample (0.5 g) by RIPA lysis buffer containing protease inhibitors (Thermo Fisher Scientific), and the protein concentrations were quantified by a BCA kit (Thermo Fisher Scientific). Deionized water was then used to dilute all protein samples (500 µg/mL), and the samples were preserved at −20 °C until used for ELISA experiments. The remaining renal tissues were stored at −80 °C in RNALater (Thermo Fisher Scientific).

Serum levels of FBG, total cholesterol, low (LDL) and high (HDL) density lipoproteins, triglycerides (TG), insulin, total proteins, albumin, urea, and creatinine (Cr) alongside spot urine concentrations of Cr and total proteins were measured on a Cobas e411 machine (Roche Diagnostics, Mannheim, Germany).

### 4.5. Quantitative RT-PCR

A QuantStudio™ 3 system was used to perform 40 amplification cycles (95 °C/15 s and 60 °C/1 min) of PCR in triplicate wells. In each well, a mixture of 5 µL SYBR Green (Thermo Fisher Scientific), 2 µL of each set of primers (5 pmol; Supplementary Table S1), and 3 µL of cDNA (25 ng) were added. Negative controls were also included and consisted of a minus-reverse transcription control from the reverse transcription step and a separate minus-template PCR, where the cDNA was substituted with nuclease-free water. *GAPDH* gene was used to normalize the results, and relative expression of mouse *TGF-β*, *iNOS*, *NGAL*, *KIM-1*, *PPARα*, *PPARγ*, and *SREBP* genes was calculated by the 2^−∆∆Ct^ method [62].

### 4.6. Immunohistochemistry (IHC)

All primary antibodies used for the detection of TGF-β, iNOS, NGAL, KIM-1, PPARα, PPARγ, and SREBP in renal tissues were mouse monoclonal IgG antibodies (Santa-Cruz Biotechnology Inc.; Dallas, TX, USA). Following blocking of endogenous peroxidases for 15 min with a BLOXALL^®^ Solution (Vector Laboratories Inc., Newark, CA, USA), the renal sections were processed with an M.O.M.^®^ (Mouse on Mouse) ImmPRESS^®^ HRP (Peroxidase) Polymer Kit to block endogenous mouse immunoglobulins (Vector Laboratories Inc). The sections were then incubated with normal horse serum for 30 min before incubation with the corresponding primary antibodies (1:200 for all antibodies) overnight at 4 °C. After washing twice with phosphate buffer saline (PBS) on the next day, ImmPRESS polymer-conjugated horse anti-mouse Ig secondary antibodies were added for 30 min (Vector Laboratories Inc). A similar protocol was also applied for the negative control slides, but with substituting the primary antibodies with primary mouse IgG isotype antibodies (Santa-Cruz Biotechnology Inc.) to control for non-specific staining, as previously described [63].

After counterstaining and cover-slipping, the sections were observed with a Leica DMi8 brightfield microscope (Leica Microsystems, Wetzlar, Germany) followed by image acquisition from 10 non-overlapping fields/section using a 40× objective. Measurement of protein expression of each targeted molecule was then conducted by the IHC Image Analysis Toolbox in the ImageJ software v 1.54f (https://imagej.nih.gov/ij/ accessed on 5 August 2023), as reported earlier [64,65].

### 4.7. Cell Apoptosis and Expression of Cleaved Caspase-3 Protein

A Click-iT™ TUNEL Alexa Fluor™ 488 Imaging Assay (Thermo Fisher Scientific) was used by following the protocol provided to detect renal cell apoptosis/necrosis. Co-expression of cleaved Caspase-3 (Casp3) protein with apoptotic bodies was then achieved by incubating the sections with M.O.M.^®^ blocking reagent for 1 h followed by adding mouse anti-Casp-3 IgG monoclonal antibodies (1:100; Thermo Fisher Scientific) for 3 h. The sections were then incubated for 30 min with donkey anti-mouse secondary IgG antibodies conjugated with a fluorescent probe (Alexa Fluor™ 555; Thermo Fisher Scientific). Following counterstaining with DAPI (Thermo Fisher Scientific), the sections were examined with a Leica DMi8 microscope at 40× magnification. Calculation of apoptosis index in each section was done by counting the percentage of dead cells in 15 fields/section, as reported earlier [66,67].

### 4.8. Western Blotting

Western blotting was used to measure SGLT2 and GLUT2 protein expression in renal tissues by mouse monoclonal IgG antibodies (Santa-Cruz Biotechnology Inc.). Briefly, renal total protein (50 μg/sample) was loaded on ready-made gradient 4–20% SDS-PAGE gels (Bio-Rad Laboratories Inc.; Hercules, CA, USA), followed by transfer to 0.45 µm PVDF membranes using a Trans-Blot^®^ Turbo™ Transfer System (Bio-Rad Laboratories Inc.). SuperBlock™ T20 blocking buffer (Thermo Fisher Scientific) was used for 15 min to block the membranes, and the primary antibodies were then added and incubated overnight at 4 °C (1:500 concentration for all antibodies). Following washing with TBS-T buffer, the membranes were incubated with secondary anti-mouse peroxidase micropolymer-conjugated IgG antibodies (1:10,000; Vector Laboratories Inc.) for 60 min at room temperature. Subsequently, SignalFire™ Plus ECL Reagent (Cell Signaling Technology Inc.) was used to develop the signals and a ChemiDoc™ XRS+ System (Bio-Rad Laboratories Inc.) was used for image acquisition. The band densitometry for each protein of interest was measured by the ImageJ software following normalisation with GAPDH protein, as reported earlier [68].

### 4.9. Enzyme-Linked Immunosorbent Assay (ELISA)

Concentrations of renal tissue TNF-α, IL-1β, IL-6, IL-10, adiponectin, leptin, and resistin were measured by mouse-specific ELISA kits (Cloud-Clone Corp.; Katy, TX, USA). Similarly, the levels of glutathione (GSH), glutathione peroxidase-1 (GPx1), superoxide dismutase-1 (SOD1), catalase (CAT), malondialdehyde (MDA), and hydrogen peroxide (H_2_O_2_) in renal tissues were quantified by ELISA (Cell Biolabs, Inc.; San Diego, CA, USA). Each renal tissue lysate sample was processed in duplicate wells using automated ELISA machine (Human Diagnostics; Wiesbaden, Germany) and by following the manufacturers’ instructions.

### 4.10. Statistical Analysis

Data analysis was performed with SPSS statistical analysis software version 25. Determination of normality and homogeneity was done by the Kolmogorov and Smirnov’s test and the Levene test, respectively. One-way analysis of variance (ANOVA) with Tukey’s HSD or Games–Howell post hoc tests were used to compare among groups based on equality of variance. Data are presented as mean ± standard deviation (SD) and *p* < 0.05 indicated statistical significance.

## 5. Conclusions

In conclusion, SGLT2i outperformed Pcal monotherapy, demonstrating greater improvements in metabolic and renal biochemical profiles, alongside better modulations of renal adiponectin, leptin, resistin, PPARα, PPARγ, SREBP-1, and markers of inflammation and oxidative stress. On the other hand, SGLT2i and Pcal co-therapy exhibited superior efficacies against DN that could be attributed to boosted remedial effects through enhanced regulation of renal glucose and lipid metabolism together with antioxidative and anti-inflammatory actions. However, more studies are needed to measure and compare the effects of SGLT2i and/or Pcal single and dual therapies on pancreatic tissues with the molecular regulatory pathways of renal glucose reabsorption, lipid metabolism, renal haemodynamics, and calcium homeostasis during the treatment of diabetes mellitus.

## Figures and Tables

**Table 1:** Body weight (mean ± SD) with serum levels (mean ± SD) of fasting blood glucose (FBG), insulin, lipid profile, total protein, albumin, urea, and creatinine alongside spot urine concentrations (mean ± SD) of creatinine, total protein, and protein/creatinine ratio in all study groups.

		NC Group	PC Group	SGLT2i Group	Pcal Group	SG-P Group
Body Weight (g) *		34.5 ± 3.4	22.2 ± 1.3 **^b^**	28.7 ± 3.6 **^b,d^**	24.8 ± 2.4 **^b,e^**	32.4 ± 2.5 **^d,e,g^**
**Serum**	FBG (mg/dL) *	81.3 ± 7.1	294.5 ± 14.6 **^b^**	127.8 ± 13.7 **^b,d^**	179.4 ± 10.9 **^b,d,f^**	100.9 ± 11.5 **^a,d,f,h^**
	Insulin (μU/mL) **	27.3 ± 5.7	5.9 ± 1.3 **^b^**	10.8 ± 2.2 **^b,d^**	8.4 ± 1.8 **^b,c^**	18.1 ± 3.2 **^b,d,f,g^**
	Total Cholesterol (mmol/L) **	1.5 ± 0.14	2.91 ± 0.09 **^b^**	2.1 ± 0.22 **^b,d^**	2.4 ± 0.25 **^b,d^**	1.71 ± 0.12 **^a,d,f,g^**
	LDL (mmol/L) **	0.37 ± 0.06	1.82 ± 0.18 **^b^**	0.92 ± 0.16 **^b,d^**	1.32 ± 0.17 **^b,d,f^**	0.67 ± 0.15 **^b,d,e,g^**
	HDL (mmol/L) **	1.1 ± 0.13	0.47 ± 0.1 **^b^**	0.78 ± 0.07 **^b,d^**	0.65 ± 0.07 **^b,d,f^**	0.96 ± 0.14 **^d,e,g^**
	Triglycerides (mmol/L) *	0.76 ± 0.12	1.73 ± 0.13 **^b^**	1.1 ± 0.15 **^b,d^**	1.35 ± 0.1 **^b,d,f^**	0.91 ± 0.09 **^a,d,e,g^**
	Total protein (g/dL) *	6.9 ± 0.87	4.46 ± 0.58 **^b^**	5.38 ± 0.53 **^b,c^**	4.92 ± 0.62 **^b^**	6.11 ± 0.7 **^d,g^**
	Albumin (g/dL) *	4.1 ± 0.7	2.3 ± 0.4 **^b^**	2.92 ± 0.39 **^b,c^**	2.7 ± 0.34 **^b^**	3.53 ± 0.45 **^d,e,g^**
	Creatinine (mg/dL) *	0.44 ± 0.1	1.25 ± 0.16 **^b^**	0.69 ± 0.11 **^b,d^**	0.82 ± 0.1 **^b,d^**	0.53 ± 0.1 **^d,e,g^**
	Urea (mg/dL) **	35.6 ± 5.1	76.2 ± 9.4 **^b^**	48.2 ± 5.7 **^b,d^**	57.4 ± 5.6 **^b,d,e^**	41.5 ± 5.4 **^d,g^**
**Spot Urine**	Creatinine (mg/dL) **	45.2 ± 7.3	22.1 ± 2.9 **^b^**	39.8 ± 4 **^d^**	36.7 ± 4.2 **^b^**	41.1 ± 3.7 **^d^**
	Total protein (mg/dL) **	4.3 ± 0.8	22.8 ± 2.7 **^b^**	14.7 ± 2.5 **^b,d^**	19.1 ± 2.4 **^b,c,f^**	7.3 ± 1.6 **^a,d,f,g^**
	Protein/Cr ratio (mg/g) **	95.6 ± 21.3	1057.1 ± 240.3 **^b^**	368.2 ± 50.9 **^b,d^**	684.3 ± 65.6 **^b,d,f^**	179.5 ± 43.5 **^b,d,f,g^**

* = Tukey’s HSD post hoc test was used following ANOVA to compare among the groups. ** = Games–Howell post hoc test was used following ANOVA to compare among the groups. ^a^ = *p* < 0.05 compared with NC group. ^b^ = *p* < 0.01 compared with NC group. ^c^ = *p* < 0.05 compared with PC group. ^d^ = *p* < 0.01 compared with PC group. ^e^ = *p* < 0.05 compared with SGLT2i group. ^f^ = *p* < 0.01 compared with SGLT2i group. ^g^ = *p* < 0.01 compared with Pcal group.

**Table 2:** Renal tissue concentrations (mean ± SD) of cytokines and oxidative stress markers in the different study groups.

	NC Group	PC Group	SGLT2i Group	Pcal Group	SG-P Group
TNF-α (pg/mL) **	29.3 ± 4.7	95.2 ± 9.9 ^**a**^	56.6 ± 8.7 ^**a,c**^	51.7 ± 8.5 ^**a,c**^	34.4 ± 3.8 ^**c,e,g**^
IL1β (pg/mL) **	24.6 ± 5.8	233.3 ± 17.2 ^**a**^	150.9 ± 16.8 ^**a,c**^	178.2 ± 16.3 ^**a,c,d**^	52.2 ± 6.1 ^**a,c,e,g**^
IL6 (pg/mL) **	29.2 ± 4.8	126.8 ± 7.9 ^**a**^	87.9 ± 10.9 ^**a,c**^	73.8 ± 7.8 ^**a,c,d**^	41.7 ± 6.5 ^**a,c,e,g**^
IL10 (pg/mL) **	51.6 ± 9.3	13.2 ± 2.7 ^**a**^	27.6 ± 6.4 ^**a,c**^	25.3 ± 3.7 ^**a,c**^	43.3 ± 5.7 ^**c,e,g**^
GSH (mg/g) *	38.3 ± 5.3	17.5 ± 5.9 ^**a**^	24.6 ± 4.8 ^**a,b**^	27.3 ± 3.7 ^**a,c**^	34.7 ± 5.2 ^**c,e,f**^
GPx1 (µg/mg) *	4.1 ± 0.8	2.1 ± 0.4 ^**a**^	2.8 ± 0.5 ^**a**^	3.0 ± 0.6 ^**a,b**^	3.9 ± 0.6 ^**c,e,f**^
SOD1 (U/g) *	45.2 ± 5.6	21.7 ± 4.5 ^**a**^	28.5 ± 4.1 ^**a,b**^	29.3 ± 4.1 ^**a,c**^	37.8 ± 3.4 ^**a,c,e,g**^
CAT (U/mg) **	253.2 ± 18.1	142.2 ± 24.4 ^**a**^	183.7 ± 22.7 ^**a,c**^	164.7 ± 12.1 ^**a**^	217.9 ± 9.9 ^**a,c,e,g**^
MDA (nmol/g) *	34.6 ± 3.8	65.1 ± 6.2 ^**a**^	53.7 ± 6.1 ^**a,c**^	52.4 ± 7.3 ^**a,c**^	38.8 ± 6.1 ^**c,e,g**^
H_2_O_2_ (μM/g) **	1.2 ± 0.2	68.2 ± 7.6 ^**a**^	28.4 ± 6.9 ^**a,c**^	25.6 ± 7.2 ^**a,c**^	4.6 ± 1.2 ^**a,c,e,g**^

* = Tukey’s HSD post hoc test was used following ANOVA to compare among the groups. ** = Games–Howell post hoc test was used following ANOVA to compare among the groups. ^a^ = *p* < 0.01 compared with NC group; ^b^ = *p* < 0.05 compared with PC group; ^c^ = *p* < 0.01 compared with PC group; ^d^ = *p* < 0.05 compared with SGLT2i group; ^e^ = *p* < 0.01 compared with SGLT2i group; ^f^ = *p* < 0.05 compared with Pcal group. ^g^ = *p* < 0.01 compared with Pcal group.

## Data Availability

The data that support the findings of this study are available within the article.

## Supplementary Materials

The supporting information can be downloaded at: https://www.mdpi.com/article/10.3390/ijms242417380/s1.
