# Supplementary material for: Improved Glycaemic Control and Nephroprotective Effects of Empagliflozin and Paricalcitol Co-Therapy in Mice with Type 2 Diabetes Mellitus
Source: Int J Mol Sci. 2023 Dec 12;24(24):17380. doi: 10.3390/ijms242417380 (PMC10743534; doi:10.3390/ijms242417380)
Supplement: Supplementary file 1 [file ijms-24-17380-s001.zip › ijms-2750546-supplementary.pdf]

Supplementary Table S1: The sequences of PCR primers used for the detection of mouse *GAPDH*, *TGF-β1*, iNOS (*NOS2*), NGAL (*Lcn2*), KIM-1 (*Havcr1*), *PPARα (PPARA)*, *PPARγ (PPARG)*, and *SREBP1 (Srebf1)* mRNAs in renal samples including the corresponding genes accession numbers and amplicon sizes.

Genes	Forward	Reverse	Amplicon size
GAPDH<br>(NCBI: NM_001289726.1)	5’ CAG GAG AGT GTT TCC TCG TCC 3’	5’ TTT GCC GTG AGT GGA GTC AT 3’	190 bp
TGFβ1<br>NM_011577.2	5’ GCC CGA AGC GGA CTA CTA TG 3’	5’ GCT TCC CGA ATG TCT GAC GTA 3’	135 bp
NOS2<br>NM_010927.4	5’ CCA CCT TGG TGA AGG GAC TG 3’	5’ AGA AAC TTC CAG GGG CAA GC 3’	165 bp
Lcn2<br>(NCBI: NM_008491.1)	5’ AGC TTT ACG ATG TAC AGC ACC A 3’	5’ TCT GAT CCA GTA GCG ACA GC 3’	108 bp
Havcr1<br>(NCBI: NM_134248.2)	5’ TGT CCC CAT GAG ACA ACA GC 3’	5’ GTT TTT CTG CGG CTT CCC TG 3’	138 bp
PPARA<br>NM_011144.6	5’ CCG AAC ATT GGT GTT CGC AG 3’	5’ AGA TAC GCC CAA ATG CAC CA 3’	161 bp
PPARG<br>NM_001127330.2	5’ GTG AGA CCA ACA GCC TGA CG 3’	5’ TCA GTG GTT CAC CGC TTC TTT 3’	110 bp
Srebf1<br>NM_011480.4	5’ CAT GAA ACC CGA AGT GGT GGA 3’	5’ CGG GCT CAG AGT CAC TAC CA 3’	125 bp
